# Mental health challenges and digital platform opportunities in patients and families affected by pediatric neuromuscular diseases - experiences from Switzerland

**DOI:** 10.1177/20552076231213700

**Published:** 2023-11-16

**Authors:** Oliver Gruebner, Afua van Haasteren, Anna Hug, Suzanne Elayan, Martin Sykora, Emiliano Albanese, Georg M. Stettner, Veronika Waldboth, Sandra Messmer-Khosla, Cornelia Enzmann, Dominique Baumann, Viktor von Wyl, Marta Fadda, Markus Wolf, Michael von Rhein

**Affiliations:** 1 Department of Geography, 27217 University of Zurich, Zurich, Switzerland; 2 Department of Epidemiology, Epidemiology, Biostatistics, and Prevention Institute, University of Zurich, Zurich, Switzerland; 3 27216 Institute of Public Health, Università della Svizzera italiana, Lugano, Switzerland; 4 Centre for Information Management, School of Business and Economics, Loughborough University, Loughborough, UK; 5 Neuromuscular Center Zurich and Department of Pediatric Neurology, 111833 University of Zurich, 30280 University Children’s Hospital Zurich, Zurich, Switzerland; 6 Institute of Nursing, School of Health Sciences, Zurich University of Applied Sciences, Winterthur, Switzerland; 7 Schweizerische Muskelgesellschaft, Zurich, Switzerland; 8 Department of Neuropediatrics, Neuromuscular Center, University Children's Hospital Basel, Basel, Switzerland; 9 Swiss Registry for Neuromuscular Disorders (Swiss-Reg-NMD), Institute of Social and Preventive Medicine, University of Bern, Bern, Switzerland; 10 Department of Psychology, 27217 University of Zurich, Zurich, Switzerland; 11 Child Development Center, University Children’s Hospital Zurich, University of Zurich, Zurich, Switzerland

**Keywords:** accessibility, digital place, disability, Duchenne muscular dystrophy, inclusion, neuromuscular disease, psychological distress

## Abstract

Receiving the diagnosis of a severe disease may present a traumatic event for patients and their families. To cope with the related challenges, digital interventions can be combined with traditional psychological support to help meet respective needs. We aimed to 1) discuss the most common consequences and challenges for resilience in Neuro Muscular Disease patients and family members and 2) elicit practical needs, concerns, and opportunities for digital platform use. We draw from findings of a transdisciplinary workshop and conference with participants ranging from the fields of clinical practice to patient representatives. Reported consequences of the severe diseases were related to psychosocial challenges, living in the nexus between physical development and disease progression, social exclusion, care-related challenges, structural and financial challenges, and non-inclusive urban design. Practical needs and concerns regarding digital platform use included social and professional support through these platforms, credibility and trust in online information, and concerns about privacy and informed consent. Furthermore, the need for safe, reliable, and expert-guided information on digital platforms and psychosocial and relationship-based digital interventions was expressed. There is a need to focus on a family-centered approach in digital health and social care and a further need in researching the suitability of digital platforms to promote resilience in the affected population. Our results can also inform city councils regarding investments in inclusive urban design allowing for disability affected groups to enjoy a better quality of life.

## Introduction

Neuromuscular diseases (NMD) belong to a diverse group of rare diseases that affect the muscles, motor nerves, motoneurons or neuromuscular junction.^1^ In addition, NMD can also be part of multisystem diseases, e.g., myotonic dystrophies or mitochondrial cytopathies.^2,3^ In most NMD, the clinical picture is characterized by muscle weakness that often progresses and may lead to motor impairment, respiratory and/or cardiac failure, and reduced life expectancy in more severe forms of NMD. One of the most common hereditary NMD is Duchenne muscular dystrophy (DMD), a severe, progressive, muscle-wasting disease, which affects almost only boys.^4^ In DMD, weakness is typically most severe in proximal muscles, e.g., pelvic and thigh muscles, and leads to gait abnormalities as an early symptom around 2–3 years of age. Due to progressive muscle wasting, most DMD patients become wheelchair dependent around age 12, and even with optimal care, mean survival in DMD is 30–35 years.^5^ Although intense research activities and several clinical trials are ongoing, no disease specific, targeted treatments (e.g., gene-based treatments) are available for DMD in Switzerland until present.^6,7^ This is true with only a few exceptions for the majority of NMD.^8–10^

Receiving the diagnosis of an NMD leading to severe disability and shortened life expectancy, as it is the case in DMD, may present a stressful or even traumatic event for the patients and their parents, and affects the entire family and its broader social system. The scientific literature on the mental health challenges and consequences of traumatic events provides robust evidence that many people show mental health resilience in this and comparable contexts.^11,12^ However, previous research has found that female family members (likely due to a higher care burden as compared to male family members), people who have pre-existing mental health conditions, and those with fewer socio-economic resources are particularly at risk of developing mental health problems in the aftermath of stressful or traumatic events.^11,13,14^ Families that show lower levels of social support may also be more likely to show less resilience.^12^ We therefore argue that such individuals may benefit from targeted support including professional services and digital interventions,^15,16^ which have shown to be effective in promoting patient self-management and improving health-related outcomes and behaviors.

In the context of DMD, psychological distress is pervasive as the muscular disease progresses over time and constitutes a constant limitation of daily living for patients and their families due to compromised motor functions. During the transition into adulthood, families are challenged by a multitude of factors, including, for example, limited access to inclusive education or the job market. In addition, family life is shaped by recurring crises and feelings of loss, grief, and fear of the future.^17^ These challenges may sum up to an increased mental health burden in families and patients as they need to adapt to the ever changing clinical (medication, treatment, doctor visits), social (friends, school, work), and physical (accessibility of the built environment) conditions. In addition, untreated mental health conditions may negatively impact the course of chronic illnesses, and may further affect all life domains, for instance, lead to lower income and lack of employment possibilities, reduced life expectancy, increased poverty, stigma, and discrimination, and higher risk for substance abuse.^18^ Also, during public health crises such as the COVID-19 pandemic, with limited mobility and socialization options due to governmental restrictions to contain the pandemic, the above-mentioned stress factors are potentially further aggravated and mental health problems increased,^19^ also due to limited access to health services, such as NMD specialists and psychologists. On the other hand, the digital transformation has also increased during the pandemic, bringing new opportunities in this context.

The digital transformation has brought innovative technologies in the clinical and public health domains, however, digital approaches such as online consultations, chatbots, or social media that systematically aim at strengthening mental health resilience of patients and families affected by NMD are still rare. In a scoping review of the recent literature, we found that digital platforms provided benefits in this context as they were used by affected family members to meet the needs for obtaining informational and other forms of social support, and as such offered increased opportunities for social inclusion via the digital domain.^20^ However, ethical and privacy concerns were raised, also showing a digital divide across socio-economic and ethnic groups.^20^

Although there is scientific evidence on specific factors causing distress in patients and family members living with a progressive neuromuscular disease,^21,22^ compared to the advancement in new digital technologies and therapies in this field of medicine, the psychosocial support has not developed accordingly.^23–28^ Furthermore, little is known about the individual needs of families with respect to coping with the challenging time during medical clarifications and after having received the final diagnosis, and factors supporting their mental health resilience.^29^ To better integrate families` needs in the context of new or adapted health- and social care interventions, we argue that digital platforms and services could be of additional benefit for social support and mental health prevention, when they provide reliable information and safe digital places for social interaction. This is particularly true for Switzerland, where to date this topic has not yet been investigated.

This perspective paper therefore aims to 1) discuss some of the most common consequences and challenges for mental health resilience in patients and family members and 2) elicit practical needs, concerns, and opportunities for digital platform use to improve mental health resilience in patients and family members with pediatric neuromuscular diagnoses.

To tackle these issues in a participatory way using an interdisciplinary and multidimensional approach that included the perspective of affected patients and their families, we pursued a two-fold strategy. First, we draw from A) an online workshop^30^ on the topic that was conducted on September 9^th^, 2021, with 15 participants who are all co-authors of this manuscript representing patient organizations, health care professionals, patient representatives, and researchers. We organized the workshop as a Zoom meeting, with keynote sessions to frame the topic, and world café and plenary sessions with a facilitated focus group discussion structure. The workshop was recorded with permissions from the individual participants. Furthermore, we took notes during the workshop and subsequently re-watched and analyzed the recordings, while any unclarity was discussed among the lead author and co-authors leading individual focus group sessions. We organized all statements in an Excel sheet according to the individual session topics. Following the rationale of Thematic Analysis^31,32^ data were organized and aggregated by three investigators (AH, AFH, OG) into themes in an iterative way until all information was processed and saturation achieved. Subsequently, consensus about the final set of themes was reached among all co-authors, that is, all participants of the workshop. Ethical approval was not required due to the co-authors all giving consent for excerpts of the workshop to be used in this paper.

Second, we draw on B) findings from the Swiss Duchenne Conference^a^ on September 9^th^ and 10^th^ 2022, where about 110 researchers, health care professionals, patients and family members, and patient organizations shared their thoughts, challenges, and opportunities in panel discussions, poster presentations, network sessions, and conference workshops. From among the authors of this paper, DB and OG participated in the conference. Regarding discussions at the conference, we presented our preliminary research from the workshop (A) in a 60-s pitch to stakeholders in the plenary session as well as in a poster session on the first day of the conference, informing them that their feedback on the data presented would be invaluable in refining, advancing, and publishing our research. Participants’ information was captured by note taking and retrospective memory notes in the aftermath of the conference. We consider the discussions that occurred in these two events A) and B) as our primary source of data, in line with Orngreen & Levinsen^33^ and Shamsuddin, Sheikh, and Keers.^34^

The following topics and issues were addressed by the workshop participants, and at the conference.

### Social and mental health challenges for patients and family members

**Figure 1.**
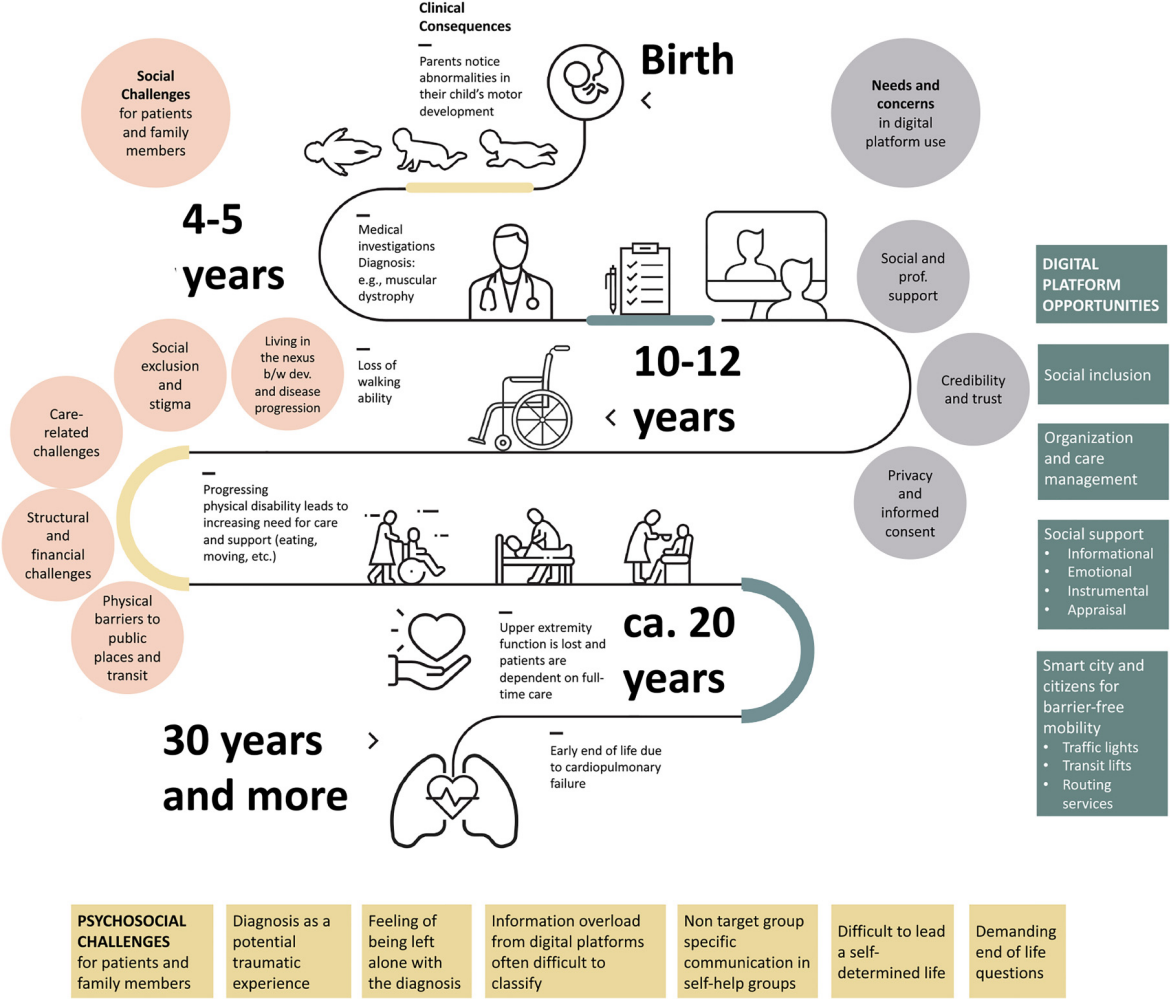
Illustrative example of the course of DMD (and similar severe, progressive muscular dystrophies / limb-girdle muscular dystrophies). The figure was produced by the authors specifically for this publication.

The progressive nature of DMD constituted by constant loss of muscular strength leads to ongoing and ever-changing challenges in the everyday lives of affected patients and their families. Based on the discussions and feedback gauged at the workshop and the conference, we have identified the following (Figure 1): Psychosocial challenges, living in the nexus between development and disease progression, challenges related to social exclusion and stigma, care-related challenges, structural and financial challenges, and challenges related to barriers in public places and transit. Participants saw these issues both with respect to disability in general, as well as within the narrower context of DMD. In addition, often even “accessible” facilities are not autonomously usable for people with DMD or comparable conditions.

#### Psychosocial challenges

At the time when parents are confronted with the diagnosis of their child, the diagnosis is likely to present a stressful or even traumatic event for parents due to the loss of hope for a ‘normal’ development and life of their child and due to being confronted with a significantly reduced life expectancy. At the conference, low barrier access to social and psychological support services was discussed as particularly crucial for the time between the first medical assessments and decisive clarifications with genetic tests. Low barrier access may reduce feelings of being left alone with the diagnosis. Although, patient organizations like Schweizerische Muskelgesellschaft (SMG)^b^ provide consultancy and guidance to find psychosocial help in this context, a repository of psychologists specifically trained for DMD-related challenges is not currently available in Switzerland. Preliminary results of CARE-NMD-CH, an ongoing project focusing on health care needs of patients with NMD in Switzerland confirmed that there is an unmet need for psychosocial assistance and social support during diagnosis and throughout the course of the disease.^35^ This conclusion was further supported in panel discussions at the conference. For example, the time in which patients experience significant physical losses, for instance, their ability to walk, was discussed as being oftentimes stressful and difficult for the entire family. Clinical guidelines for diagnoses and management of DMD recommend mental health assessment of patients and family members and provide psychosocial care where needed.^4,36,37^ However, adherence to clinical recommendations varies across diverse health care services in Switzerland due to health insurance related and socio-demographic factors and further includes provider and patient preferences as found in previous studies.^38,39^ This may also be true for psychological health care utilization in the context of DMD and should be further explored in future studies.

#### Living in the nexus between development and disease progression

Life cycle transitions pose an extraordinary challenge for people and families affected by DMD. The tension between patients’ development into independent individuals and their progressing muscular degeneration leading to increased physical dependence has potential consequences for mental health of both patients and the entire family, including parents, grandparents, and siblings, as was discussed in the workshop and conference. Others have also found that these stressors may be particularly important during the time when walking ability is lost, and at the end of life.^22^ In this context, Waldboth *et al.*^17^ have developed an interpretative model that identifies four domains in family life that are of particular importance when considering mental health stressors. While the relational domain (experiencing closeness and distance) was less discussed at the workshop and conference, the social (adapting to social norms while facing increasing level of physical impairments), the functional (living with physical dependence vs. striving for independence), and the emotional domains (sadness and fear versus being full of hope, psychosocial challenges) were more prominent in the discussions around social and mental health challenges.

#### Challenges related to the social domain: exclusion and stigma

Low levels of integration in the social domain, such as in childcare, kindergarten, school-, or work contexts were additional challenges for children and family members mentioned during the workshop and the conference. In this context, there is the risk that children feel or are excluded from social activities when they cannot keep up with the pace of their peers due to their decreasing muscle function, especially when respective assistance personnel are not available. This can have negative consequences for mental health, or quality of life (e.g., developing low self-esteem or feeling excluded or isolated). According to the participants of the workshop and discussions at the conference, following the daily schedule in kindergarten or schools often becomes increasingly impossible for affected children, as changing times before and after sport lessons may be too short to successfully be managed by the child alone. In Switzerland, personal assistance is usually available to help the affected child through their daily activities at kindergarten, or at school. However, some parents experience the process of initiating assistance and continuously organizing substitutes if one assistant is not available anymore, as stressful. In addition, based on their statements, some parents experience a gap between the theoretically granted support in Switzerland, and the real situation, with a shortage of available aides. To achieve a proficient level of integration into the social environment for their affected child (e.g., personal assistance) was described as an oftentimes high logistical burden and as a stressful, sometimes even frustrating activity. However, if personal assistance is available, this was seen as extremely supportive to manage the daily activities between kindergarten or school and routine weekly consultations with service providers such as speech therapy, occupational therapy, or physiotherapy.

Furthermore, there is the risk that the affected child is stigmatized in their social environment as the one that constantly needs assistance, which may additionally be experienced as stressful. Patient organizations like SMG offer much needed counseling and educational training for teaching personnel and students in schools to help reduce stigmatization and improve inclusion from early disease stages on. However, the state, the education system, and the schools are required to proactively support affected children to guarantee a successful inclusion,^40^ art. 24, a-c.

#### Care-related challenges

With respect to the functional domain, physical dependence on others was reported as a challenge. Depending on the degree of disability, family situation, and personal attitude of the involved persons (patient and relatives), workshop participants reported a high care burden and that care related stressors can get overwhelming as they converge and accumulate over time. The CARE-NMD-CH study mentioned earlier confirmed that in general, affected families have a moderate burden of care in Switzerland rated on a scale from 0–30 with moderate 12.6 (standard deviation = 8.5) points.^17^ The study revealed that 60% of participating parents gave dedicated support and 90% accompanied their affected family members on a regular basis to health services.^17^ Organizing care for patients with severe physical disability, who are increasingly dependent on the help of others and therefore must carefully plan their everyday life, was considered as a stress factor for them and their families in their struggle to maintain a self-determined life. Also, more equally distributing the burden of care among different family members, or professional assistance seemed to reduce involved stressors for patients and the entire family. A better distribution of the care burden seems to be even more substantial when patients have siblings.

Further stressors mentioned in the context of external home care include that those services are provided by ambulatory chronic care personnel, such as the Swiss home care nursing service called “Spitex”, for which personnel is usually available during office hours and hence does not necessarily fit the needs of a working patient or their working parents. Situations in which patients or their families have to adapt to service times of the external care provider were reported as highly unsatisfactory. Also, an often-reported issue with external home care providers was the frequent turnover of personnel, where additional time and effort is required to brief and train fresh staff members, especially with regards to patient specific needs and requirements.

Another issue that was highlighted at the workshop and conference was the perceived lack of access to psychologists specialized in caring for patients with complex needs. This issue was seen as a shortcoming for the patients and families, especially at critical timepoints such as during times of diagnosis, when patients lose their walking ability, and during end of life. Relatedly, lack of specialization on chronic physical conditions and complex mental health conditions in psychologist training has been identified in the past also by other scholars.^41,42^ At the conference however, conversations around psychosocial stressors affecting patients and families tended to agree that low-threshold psychological care can already mitigate the mental burden in the affected population group, even when psychologists are not specifically trained in rehabilitation psychology for the context of chronic/progressive conditions and limitations of (motor) functions. Furthermore, it also has to be stressed that readiness for psychological support and palliative care (PC) services may be at stake, that is, some family members and affected individuals may not want to get psychological or PC support due to stigma, which would again call for low-threshold, prevention, or health educational interventions.

#### Structural and financial challenges

Structures within the Swiss health and social care system are not yet fully connected in a way that guarantees smooth clinical transitions and effective information exchange. This lack of intersectional connectedness creates a continuous and distressing urge for parents or caregivers to report on the disease history of their children repeatedly at first encounter visits. This situation was considered particularly stressful if younger affected children had to accompany the visit and are present during the consultations, listening to the conversation between the parents and the case manager or physician.

Workshop and conference participants also agreed that although the Swiss social security system provides a high level of support, chronic disabilities are associated with financial challenges that are not fully compensated. One of the points made in workshop discussions was that the financial costs associated with disability are an additional stressor for patients and families. Examples given included parents having to repeatedly argue with insurance companies to ensure that costs are covered or that they are not covered at all. In Switzerland, a national disability insurance (in German: Invalidenversicherung or IV) takes over most disability-related costs once a genetic NMD has been conclusively confirmed. This is true until the patient reaches the age of 20 years, then the national mandatory health insurance takes over the costs thereafter. However, this only affects individuals with a social security number in Switzerland. Furthermore, affected families are sometimes not aware that the IV is taking over relevant costs, unnecessarily adding to the financial burden of the family. In this regard and many other regards, low-threshold non-profit counseling services on financial support such as the ones offered by SMG and other patient organizations such as ASRIMM (Association Suisse Romande Intervenant contre les Maladies neuroMusculaires) or MGR (Associazione Malattie Genetiche Rare Svizzera italiana) provide professional advice offered at no costs.

#### Physical barriers to public places and transit

Workshop and conference attendants identified the non-inclusive urban design as a major concern with architectural barriers posing substantial logistical problems for self-determined mobility of disability-affected persons. Specifically, the built environment was considered as not always suited to complying with specific health care needs, or impairments (e.g., immobility). Public and private buildings, places of everyday activities, and public transport were reported to be hard to access, sometimes even health care facilities were reported to be poorly accessible. A finding that has also been confirmed in the literature as e.g., urban areas have been traditionally designed for the ethnic majority, able-bodied, working-aged people^43^ and therefore rarely provide access for disability affected persons and other groups.^44–50^ Other researchers report similar structural inaccessibility such as Gleeson^51^ who examined how disabled people's lives are made more challenging by contemporary urban planning and architecture, which often do not consider the needs of disabled people. In 2007, Imrie and Edwards conducted a review and found that human geography rarely engages with disability,^52^ however more recently Hoskin^53^ argued that this is very country specific. Furthermore, workshop participants mentioned that mobility in the public domain may be impeded by communication barriers, such as speaking problems due to disability or being shy or reluctant to clearly express the need for help to strangers. Particularly for Switzerland, the United Nations Committee on the rights of persons with disabilities in their concluding observations on the initial report,^40^ art. 9, a noted with concern “*The lack of a comprehensive accessibility strategy to harmonize accessibility obligations at the federal, cantonal and municipal levels, to embed universal design standards and to encompass all domains, including public transport, buildings, facilities, public spaces, services, and physical, information, communications and digital access, and including at the design and construction phases*”. For this reason, the committee recommended to “*Adopt an accessibility strategy, in close consultation with organizations of persons with disabilities, to harmonize accessibility across all levels of government, to embed universal design standards and to ensure access to all domains*”. Our findings may help inform city governments about the need to invest more in inclusive urban design to create a better quality of life for groups affected by disability.

In addition, participants pointed to reservations of landlords towards handicapped tenants leading to a partial exclusion from the free market of living space. Our study also found undersupply of barrier-free payable living space and difficulties in dynamically reacting to changing needs and impairments of the affected children along with financial challenges that emerged as issues that created psychological and emotional distress. For example, when entrance areas or sanitary facilities in rented flats need to be restructured to facilitate a barrier free access, house owners need to grant permission and financial resources need to be available. In Switzerland, for example, the Swiss Agency for Obstacle-Free Architecture^c^ provides guidance and support in this context.

### Needs, concerns, and opportunities in digital platform use

#### Social and professional support through digital platforms

Key features of digital platforms identified during the workshop included interaction and social exchange options (e.g., peer-support) for the patients, parents, and the entire family to provide emotional support and appraisal. In this way, emotional support could be gathered through individual friends’ networks on social media but also from other affected families and patients through messaging services and group chats on popular social media platforms. Other scholarly work provides evidence that digital platforms are most effective and appropriate if they are used to connect patients and families amongst each other in a safe space for private communication and exchange.^54^ For the workshop participants, this was also true in the Swiss context. At the conference however, self-help groups sponsored by SMG were predominantly used by parents and other family members, but less so by the patients themselves, potentially due to the reason that individuals with DMD do not always want contact with other affected individuals only but rather prefer inclusive settings, which are open to non-affected individuals. In this line, gaming platforms were more frequently mentioned as patients enjoyed these platforms to navigate freely in the digital sphere without barriers and without possible stigma related to physical disability.

Furthermore, instrumental support, that is, tangible aid through apps and other digital platforms were mentioned as useful and desired by participants. These digital platforms could include organizational tools for everyday planning, medication intake management targeted directly at (pediatric) patients, as well as further tools to help bridge and mitigate frequent turnover of medical personnel. Also, navigation tools combined with e.g., a geo-repository or routing options for user-rated accessible sites such as those (to some extent) implemented in the Ginto^d^ or CaptureAndGo^e^ applications were considered to be useful in this context. Workshop participants provided anecdotal evidence that disability-affected persons have difficulties navigating freely in the urban context due to physical and other architectural barriers. In this regard, opportunities emerge from Smart City and Internet of Things (IoT) applications.^55^ IoT applications may allow for a more equitable and inclusive urban design providing possibilities to engage with the environment via sensors and digital devices. For example, street features such as traffic lights may be equipped with an interface or programmed so that they become aware of people with specific needs such as persons with reduced mobility (e.g., wheelchair users) to give them more time to cross the streets, while slowing down motorized individual traffic adequately. Another example includes elevators in train stations or public buildings, which could be equipped with sensors that enable coupling via digital devices. This would allow people with motor disabilities to use elevators or traffic lights more independently without having to press a physical button, and this enables for more inclusion of disability affected persons in public places, buildings, and transit. In addition, smart citizen applications such as the Ginto or CaptureAndGo applications allow for users to rate individual physical places in relation to their accessibility and such help to create a voluntarily shared and updated knowledge base to increase barrier-free mobility. In sum, with the help of digital technologies, navigating through the city could become a safer and less stressful experience for individuals with special needs, and the urban environment in general could become more inclusive and reliable for everyone.

#### Credibility and trust

Workshop participants discussed that more credibility and trust in online information, particularly for user-generated content, is needed. This need has also been confirmed in a scoping review of the scientific literature.^20^ There is a clear demand for verified, centralized, and continuously updated information on the disease and its development in the body and mind over time, on possible treatment options, and on sources of support. Digital platform applications should further allow for an easy discovery of reliable psychosocial support-related resources, when needed (e.g., a registry with specialized professionals and therapists), that is, provide easily accessible informational support. In this context, workshop participants identified five fields of improvement for digital platforms aiming to provide informational support: 1) Hands-on information about specific needs should be gathered directly from the affected persons in systematic ways, 2) information should be tailored to the needs of different groups (e.g., groups in which the disease had been recently diagnosed versus groups in which the disease has progressed to particular stages), 3) information should not only be targeted at parents but also directed at children and individuals in different age groups, 4) information should be communicated with respect to cultural, age-specific, and language barriers. Finally, 5) contact information on professionals/therapists, specialized centers, and other helpful resources should be shared.

However, such platforms are not yet commonly offered and hence, social media platforms are often used such as Facebook groups or WhatsApp chat groups, to organize care and several types of social support. Ideas for more formalized solutions of more integrated digital platforms included A) a market space for quick and just-in-time help, B) a geo-registry for accessible and specialized professionals/therapists with the option to offer also online consultations to bridge geographical gaps in service provision, and C) structured information on financial aid possibilities. Furthermore, there is an opportunity and need for target group specific design to guarantee low barrier digital access to these platforms.^56^

#### A central feature: privacy and informed consent

Overall, privacy has been highlighted repeatedly in the scientific literature as a substantial and key concern when using digital platforms, especially given the sensitive and private nature of support interactions and patient care-related exchanges.^57–59^ However, this topic did not seem to play a major role in patients and their families when they choose digital platforms for social support, according to discussions at the workshop and conference. One concern here, particularly with private digital platforms, was the perceived lack of informed consent due to notoriously lengthy and unclear policy declarations of many digital platforms.^60^ As highlighted by Reinhardt *et al*.,^61^ “*making users aware of the data policies of an organization to let them choose which of these policies they are willing to consent to*” is considered a key privacy mechanism of a digital platform. Hence, meaningful innovative approaches, such as privacy labels for digital content as proposed by Kelley *et al*.,^62^ and more recent interactive versions of it,^61^ might provide for a more appropriate, transparent, and clear process for informed consent. Alternative solutions that ensure users’ privacy in digital platforms are needed,^60^ to ensure that the user has primary control regarding how, where, when, and what personal information is used for what purposes, and for how long such information is retained. Such standards of data collection and processing (including data processing by third parties and partners with whom data are shared) can only be achieved by respective legislative regulations. On the other hand, in some instances privacy is less desired, and in our recent scoping review of digital platform use^20^ we found that one of the reasons for their utilization was, for instance, advocacy, with a popular public platform of choice, where a more public facing set of platform affordances might be preferable. A study by Bussone *et al*.,^63^ found that patients were acutely aware of their requirements for robust privacy and security when their data is being handled. Although the patients in their study indicated a willingness to share some of their digital identity attributes, such as gender, age, medical history, health, and well-being data, they were not willing to disclose details that could reveal their personal identity.

## Conclusions

Following an insightful online workshop that included 15 participants consisting of health care professionals, affected family members, patient organization representatives, and researchers as well as a conference where about 110 participants including researchers of DMD, practitioners, patients, and family members shared their thoughts, challenges, and opportunities in panel sessions and workshops, we conclude that there is an urgent need to focus on a family centered approach in health and social care. Furthermore, there is a need for research on the suitability of digital platforms to foster and promote resilience in the DMD affected patients and their families. The needs of DMD patients may be quite specific, and their coping strategies are different from mental health patients who are not affected by chronic or progressive conditions. However, although we identified these needs within the context of DMD, we see our findings as potentially applicable to many other chronic and disability affected health conditions.

Our discussion panels highlighted several factors, such as the risk of affected individuals and their family members of developing mental health problems that require the attention of health care professionals. Particularly, targeted access to psychologists and to other service providers who specialize in chronic or progressive conditions and limitations of motor functions is required. Discussions during the conference and workshop also identified the need for safe, reliable, and expert-guided digital information and platforms to become more readily available. These digital platforms could include optional online access to mental and/or physical healthcare providers, facilitate networking and coordination of care to enable ongoing professional and peer-support, assessment, and monitoring of the lives and health of patients and their families. Such platforms could also provide navigation and way finding applications to aid in accessibility of routes, allowing users to rate accessible facilities. Psychosocial and relationship-oriented family digital interventions are warranted, to provide care and support for patients and their families and offer easier access to healthcare service providers. City and town councils need to invest in inclusive and accessible urban architecture taking advantage of smart city applications to create a safe and navigable urban environment allowing for disability affected groups to enjoy a better quality of life. We highlighted some of the consequences and challenges that those affected with DMD and their families face and elicited needs, concerns, and opportunities for digital platform use in this context. We thereby hope to help pave the way for future researchers, urban designers, information management experts, and policy makers to create more inclusive physical (and social) environments and design digital platforms that could aid in reducing some of the barriers identified in Switzerland and in comparable settings worldwide.

## Abbreviations

ASRIMM: Association Suisse Romande Intervenant contre les Maladies neuroMusculaires
DMD: Duchenne Muscular Dystrophy
IV: Invalidenversicherung
NMD: Neuromuscular Disorder
MGR: Associazione Malattie Genetiche Rare Svizzera italiana
SMG: Schweizerische Muskelgesellschaft

## Notes

a. https://progena.ch/duchenne-konferenz-2022/

b. The Schweizerische Muskelgesellschaft (SMG) (engl. Swiss Muscular Society) provides advice and training on social security and legal issues, promotes patients’ self-determination, and offers financial support. https://www.muskelgesellschaft.ch

c. The Swiss Agency for Obstacle-Free Architecture (Schweizer Fachstelle Hindernisfreie Architektur) provides consulting and specialized information about the legal regulations for everyone: https://hindernisfreie-architektur.ch

d. https://www.ginto.guide

e. https://apps.apple.com/se/app/captureandgo/id1576200735
